# Impact of age on central lymph nodes involvement in papillary thyroid cancer

**DOI:** 10.1186/s12885-024-12198-6

**Published:** 2024-04-05

**Authors:** Shadi Awny, Ahmed Abdallah, Islam H Metwally, Khaled Abdelwahab, Mohammad Zuhdy, Omar Hamdy, Ahmed M Fareed, Khalid Atallah

**Affiliations:** Surgical Oncology Department, Oncology Center Mansoura University (OCMU), Mansoura, Egypt

**Keywords:** Papillary thyroid cancer, Central nodes, Prognosis, Staging

## Abstract

**Background:**

Total thyroidectomy is the main line of treatment for papillary thyroid cancer. Central lymph node dissection (CLND) is still debatable. In this study, we aimed to correlate the central lymph node status with the age of patients.

**Methods:**

This is a retrospective study including patients with papillary thyroid cancer (PTC) who underwent total thyroidectomy and CLND at a tertiary cancer center during the period from January 2012 to September 2022. Patients were subdivided into 3groups: patients younger than 20 years old, patients between 20 and 40 years old, and patients older than 40 years old. Correlation between central lymph node status, lateral lymph node status, and harvest count with each other and between age groups was done.

**Results:**

315 patients were included. The younger the age group the higher the possibility of harboring positive central nodes, however, the positivity of lateral nodes was similar. Neither central nodal harvest nor positive central node count significantly differed between groups. The lateral nodal harvest was significantly higher in the < 20 years group with no affection to the number of positive nodes retrieved. The younger the age group the longer the disease-free survival (DFS).

**Conclusion:**

We can conclude that patients younger than twenty years had a higher probability of harboring malignancy in central nodes and higher lateral node harvest on dissection. In contrast, they do have a lower incidence of recurrence.

## Background

Thyroid cancer is the most common endocrine malignancy worldwide [1]. It accounts for 1% of all cancers [2]. Papillary thyroid cancer (PTC) accounts for approximately 90% of all thyroid cancers. Its incidence has been significantly increasing in the last decades [3].

PTC has an excellent prognosis, and the 5-year survival rate of those patients is generally above 97% [4]. It also has a much poorer prognosis in elderly people, although the reason for this finding has not been clearly identified [5]. Unlike other cancers, age is a part of the staging of PTC, the age cut-point was increased from 45 to 55 years in the 8th edition American Joint Committee on Cancer (AJCC) staging system for differentiated thyroid cancer (DTC), Young and middle–aged PTC patients are classified into stages I and II, regardless of local extension and metastasis [6].

Age is considered one of the most principal factors for determining further therapeutic strategies for patients with PTC. In addition to age, large tumor size, lymph node metastasis (LNM), and distant metastasis have a poor prognosis in DTC [7].

Central cervical lymph node metastases (CLNM) can be found in 40–60% of cases [8] Unfortunately, lymph nodes in the central neck compartment are more difficult to image via ultrasound when compared to the lateral neck compartment [9].

According to the data from Liu Y et al., the presence of CLNM and the number of metastatic cervical lymph nodes are associated with compromised survival. LNM carries a poor prognosis and shorter overall survival (OS) in PTC. However, LNM incidence is high while mortality is low in young patients. Therefore, the relationship among age at diagnosis, LNM, and OS is still inconclusive [10].

We hypothesized that the rate of CLNM increases with a younger age population. To assess this, we compared the rate of lymph node metastasis in young children & and adolescents (0–20 years), and young adults (21–40 years) with patients aged > 40 years, using a population-based data set.

## Patients and methods

This is a retrospective cohort study. We included patients with PTC who were managed by total thyroidectomy with central lymph node dissection (CLND) with or without lateral lymph neck dissection (LLND) at the time of diagnosis at the Oncology Center Mansoura University from January 2012 to September 2022. A total of 324 patients were assessed. Patients who underwent CLND in recurrent settings and those in whom central dissection did not reveal nodal tissue were excluded. Nine patients were excluded, so finally 315 patients were enrolled in the study. Demographics, preoperative, operative, postoperative, pathologic, and oncologic follow-up data were retrieved from a prospectively maintained electronic database.

According to age, patients were subdivided into three main age groups, the first group included patients younger than 20 years old, the second group included patients between 20 and 40 years old, and the third group included patients older than 40 years old.

We obtained approval from the Institutional Review Board (IRB) of the Mansoura Faculty of Medicine with code R.22.10.1875.

### Preoperative investigations

Neck ultrasound (US), thyroid function tests, and fine needle aspiration cytology (FNAC) were done in addition to routine preoperative investigations for all patients. Neck US was conducted at the first presentation to assess thyroid nodules regarding the number, size, site, multicentricity, and degree of suspicion and also to assess the central and lateral lymph nodes status. The median interval time between neck US and surgery was 3 weeks. FNAC was interpreted by the pathologists using the Bethesda classification.

### Operative technique

All operations were performed by surgical oncologists with various levels of experience and consisted of a total thyroidectomy with at least CLND through a Kocher incision; the infrahyoid muscles were opened along the midline and muscles were divided, as necessary. In all patients, every effort was made to identify and preserve the recurrent laryngeal nerves (RLN) and the four parathyroid glands. Prophylactic CLND was performed in patients with PTC with clinically node-negative disease, mainly in the ipsilateral compartment. Therapeutic CLND was performed when abnormal lymphadenopathy was detected during the preoperative or intraoperative examination. Lymph node sampling was also performed in some patients with clinically node-negative disease. LLND was performed only in patients with radiologically &/or clinically suspicious lymph nodes in the lateral neck, including levels II–V.

### Follow-up

Postoperative follow-up included postoperative complications (mainly RLN injury and hypocalcemia) in addition to disease recurrence, disease-free survival (DFS), and OS. Regarding complications, vocal fold function was assessed in all patients by laryngoscopy examination before and after surgery. RLN injuries were defined as dysfunction or total absence of vocal cord mobility compared to the contralateral one based on preoperative fiberoptic laryngoscopy. All vocal fold palsies lasting for more than 6 months were considered permanent. Postoperative serum calcium levels were measured only in patients with symptoms of hypocalcemia. All patients were also systematically examined postoperatively for other relevant complications such as hematoma, chyle leakage, and nerve damage (spinal accessory nerve, vagus nerve, phrenic nerve, and/or sympathetic trunk).

Follow-up was done for those patients regarding the oncologic outcome, recurrence, and patterns in addition to DFS and OS.

### Statistical analysis

We use the statistical software SPSS (Statistical Package for Social Scientists SPSS 26; Armonk, NY: IBM Corp) to analyze the study results. Continuous variables are presented as mean and standard deviation if normally distributed or median and range when non-normally distributed. The Mann-Whitney U test was used to compare non-parametric data. Categorical data were compared by Pearson’s Chi-square test or Fischer-Exact test as appropriate. Normality was tested by the Kolmogorov-Smirnov test. Disease-free survival was measured from the date of operation using the Kaplan-Meier curve and significance was measured using the log rank test. A p-value of ˂0.05 is considered statistically significant.

## Results

Three hundred twenty-four patients were recruited. Nine patients were excluded because the central node harvest was zero, so finally 315 patients were included in the analysis.

### Epidemiologic, operative, and pathologic criteria (Table 1)

**Table 1:** Basic epidemiologic, operative, pathologic, and complications in recruited patients

Variable	Value
Age at surgery mean +/-SD (range)	39.4 +/-14.5 (8–82) years
Sex Male Female	81 (25.7%) 234 (74.3%)
BMI median (range)	31.4 (17.5–56.9) Kg/m^2^
Radiologic central nodes Negative Positive	287 (91.1%) 21 (6.7%)
Radiologic lateral nodes Negative Positive	161 (51.1%) 148 (47%)
Surgery Ipsilateral central Bilateral central Ipsilateral central & lateral Bilateral central & ipsilateral lateral Bilateral central & lateral Ipsilateral central & bilateral lateral	102 (32.4%) 47 (14.9%) 91 (28.9%) 34 (10.8%) 30 (9.5%) 11 (3.5%)
Pattern of node dissection Prophylactic Therapeutic Sampling Excised with specimen	108 (34.3%) 146 (46.3%) 39 (12.4%) 15 (4.8%)
Operative time median	180 (60–600)
Postoperative hypocalcaemia No Yes	255 (81%) 60 (19%)
Postoperative nerve injury No Yes	285 (90.5%) 29 (9.2%)
Pathology tumours size median (range)	2.5 (0.2-8) cm
Pathologic focality Unifocal Multifocal	157 (49.8%) 145 (46%)
Pathologic laterality Unilateral Bilateral	206 (65.4%) 98 (31.1%)
Extrathyroid extension** No Yes	172 (54.6%) 83 (26.3%)
Central node harvest median (range)	5 (1–35)
Central node status Negative Positive	108 (34.3%) 203 (64.4%)
Lateral node harvest (range)	19 (1–98)
Lateral node status* Negative Positive	20 (12.1%) 145 (87.9%)
T stage 1 2 3 4	116 (36.8%) 100 (31.7%) 69 (21.9%) 15 (4.8%)
N stage 0 1	94 (29.8%) 218 (69.2%)
Recurrence No Yes	280 (88.9%) 29 (9.2%)
Pattern of recurrence* Local Nodal Distant Local & distant Local, regional & distant	2 (6.9%) 19 (65.5%) 3 (10.3%) 3 (10.3%) 2 (6.9%)

*Valid percent

**Some data are missing

The mean age was 39.4 years. Female preponderance was noted (74.3%).

All patients underwent CLND; however, the commonest pattern of node dissection was ipsilateral CLND (32.4%) followed by ipsilateral central & and lateral (28.9%). In about half (46.8%) nodal dissection was therapeutic. The median operative time was 3 h.

Postoperative symptomatic hypocalcemia affected 19% (78.3% of them were temporary). In addition, RLN injury was noticed in 9.2% (of them 48.1% were permanent, but at least two patients of them nerve was intentionally sacrificed).

Pathologic tumor median size was 2.5 cm, half (49.8%) was unifocal, and about 2/3 (65.4%) unilateral. Extrathyroidal extension was reported in 26.3%. Two-hundred sixteen (68.5%) of the patients had pathologic T1-2 tumors. More than two-thirds (69.2%) of the patients had nodal deposits (N1). Central nodes were positive in 64.4% of while lateral nodes were positive in 46% of the patients (actually representing 87.9% of those who underwent lateral dissection). In addition, positive lateral nodes were almost always associated with positive central nodes (127 out of 145 patients) (*p* =.001).

**Fig. 1:**
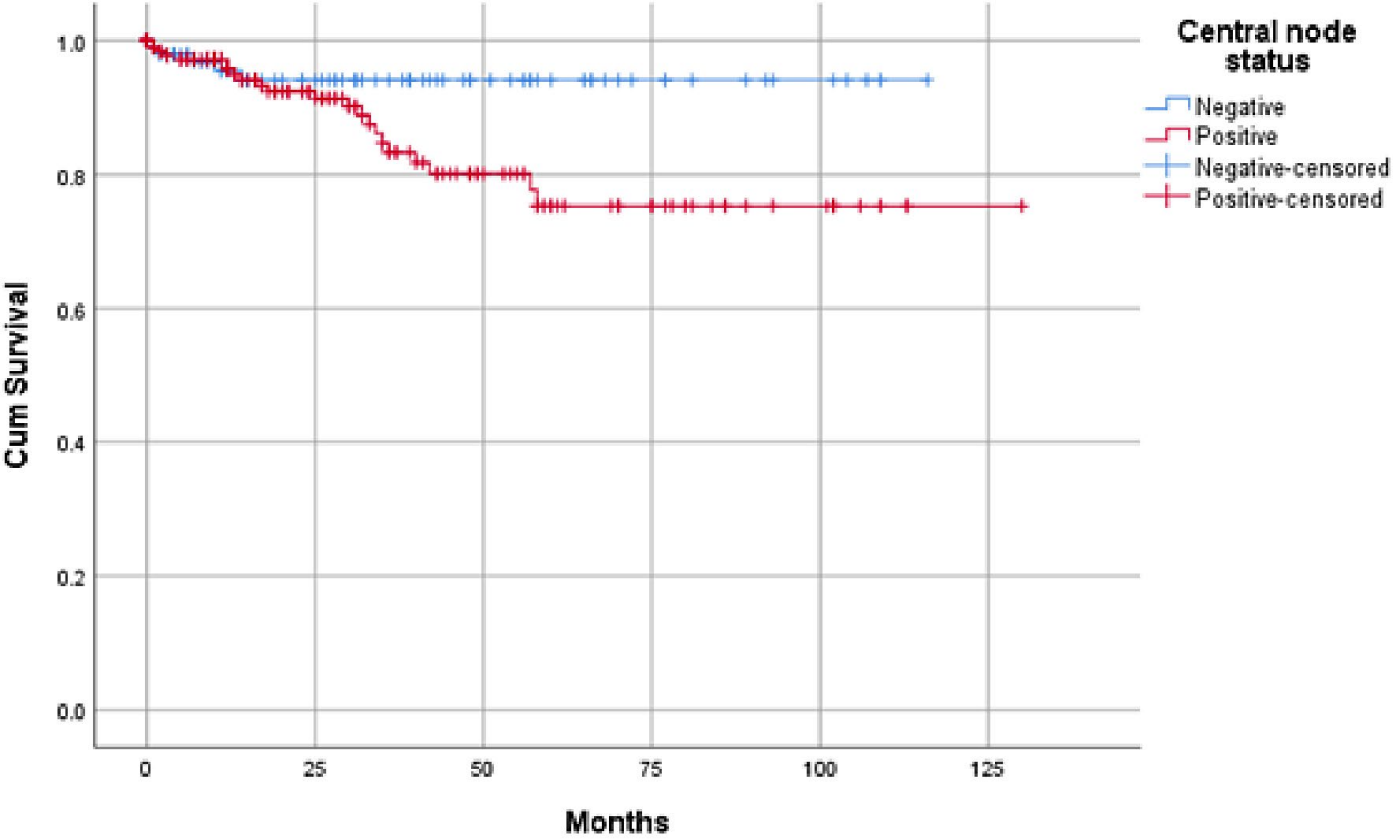
Kaplan-Meier curve showing disease-free survival according to central nodal status

226 (71.7%) out of the included 315 patients receiving adjuvant RAI. Recurrence occurred in only twenty-nine patients (9.2%) and was mostly nodal (65.5%). Also, those with positive central lymph nodes showed worse DFS (estimated mean 105.7 VS 109.6 months); however, this did not reach significance (*p* =.053) (Fig. 1). The mean follow-up time was 32.03 months.

#### Correlation with radiology (Table 2)

**Table 2:** Sensitivity, specificity, and imaging accuracy in detecting central and lateral nodal disease

Variable	Sensitivity	Specificity	Accuracy
Bilaterality	69.8%	84.8%	79.9%
Central node metastasis	7.5%	94.3%	37.5%
Lateral node metastasis	88.8%	60%	85.3%

The sensitivity of US for central nodes was 7.5% with a specificity of 94.3% and an accuracy of 37.5%. While for lateral nodes sensitivity was 88.8% and specificity 60%; Accuracy was 85.3%.

#### Comparing age groups (< 20 years, 20–40 & >40) (Table 3)

**Table 3:** Comparison of epidemiology, pathology, and outcomes among age groups

Variable	Group I (≤ 20y) 27 ptn	Group II (> 20-40y) 146 ptn	Group III (> 40y) 142 ptn	Significance
Sex Male Female	8 19	31 115	42 100	0.24
BMI median (range)	25.1 (17.5–37.5)	31 (19.5–46.7)	34 (20.5–56.9)	**< 0.001**
MNG association No Yes	18 8	85 56	55 84	**< 0.001**
FNAC (Besthesda) I II III IV V VI	0 0 0 5 10 7	7 3 5 25 65 15	0 9 9 12 64 28	**0.003**
Operative time median (range)	180 (90–540)	180 (60–600)	180 (60–510)	0.3
Surgery Ipsilateral central Bilateral central Ipsilateral central & lateral Bilateral central & ipsilateral lateral Bilateral central & lateral Ipsilateral central & bilateral lateral	6 5 6 4 5 1	50 22 41 19 11 3	46 20 44 11 14 7	0.54
Tumour size median (range)	2 (0.5-8)	2 (0.4-6)	2.5 (0.2-7)	0.19
Pathologic focality Unifocal Multifocal	17 10	76 65	64 70	0.29
Pathologic laterality Unilateral Bilateral	20 7	94 47	92 44	0.75
Extrathyroid extension No Yes	16 9	81 27	75 47	0.08
Central node harvest median (range)	6 (1–31)	6 (1–23)	5 (1–35)	0.12
Central node status Negative Positive	3 24	47 96	58 83	**0.009**
No of + ve central nodes median (range)	4 (1–17)	3 (1–20)	3 (1–15)	0.27
Lateral node harvest median (range)	24 (3–98)	16 (1–64)	21 (2–73)	**0.004**
Lateral node status Negative Positive	2 15	9 63	9 67	0.99
No of lateral + ve nodes median (range)	8 (2–43)	5 (1–16)	4 (1–24)	0.22
T stage 1 2 3 4	10 10 6 1	59 50 22 5	47 40 41 9	0.14
N stage 0 1	3 24	44 99	47 95	0.07
Postop hypocalcaemia No Yes	22 5	117 29	116 26	0.94
Postop nerve affection No Yes	25 2	138 8	122 19	0.061
Length of hospital stay median (range)	3 (1–10)	2 (1–20)	2 (1–25)	**0.013**
Recurrence No Yes	25 1	135 8	120 20	**0.026**
Estimated mean disease-free survival (95%CI)	108.6 (100-117.1)	106.4 (99.9-112.9)	101.8 (89.8-113.8)	**0.043**

There was no difference in sex distribution, operative time, type of surgery, pathologic tumor size, pathologic focality, laterality, T or N staging, extrathyroidal extension, or postoperative complications (hypocalcemia and nerve injury) among groups.

The BMI was significantly higher in elder groups (p = < 0.001),

The PTC was associated with other goitrous nodules (MNG) mainly in the group > 40 years old (*p* <.001), thus FNAC has had a significantly higher diagnostic value in the young < 20-year group (*p* =.003).

The younger the age group the higher the possibility of positive central nodes (*p* =.009), however, the positivity of lateral nodes was similar (*p* =.99).

Neither central nodal harvest nor positive central node count significantly differed between groups (*p* =.12 and 0.27, respectively).

**Fig. 2:**
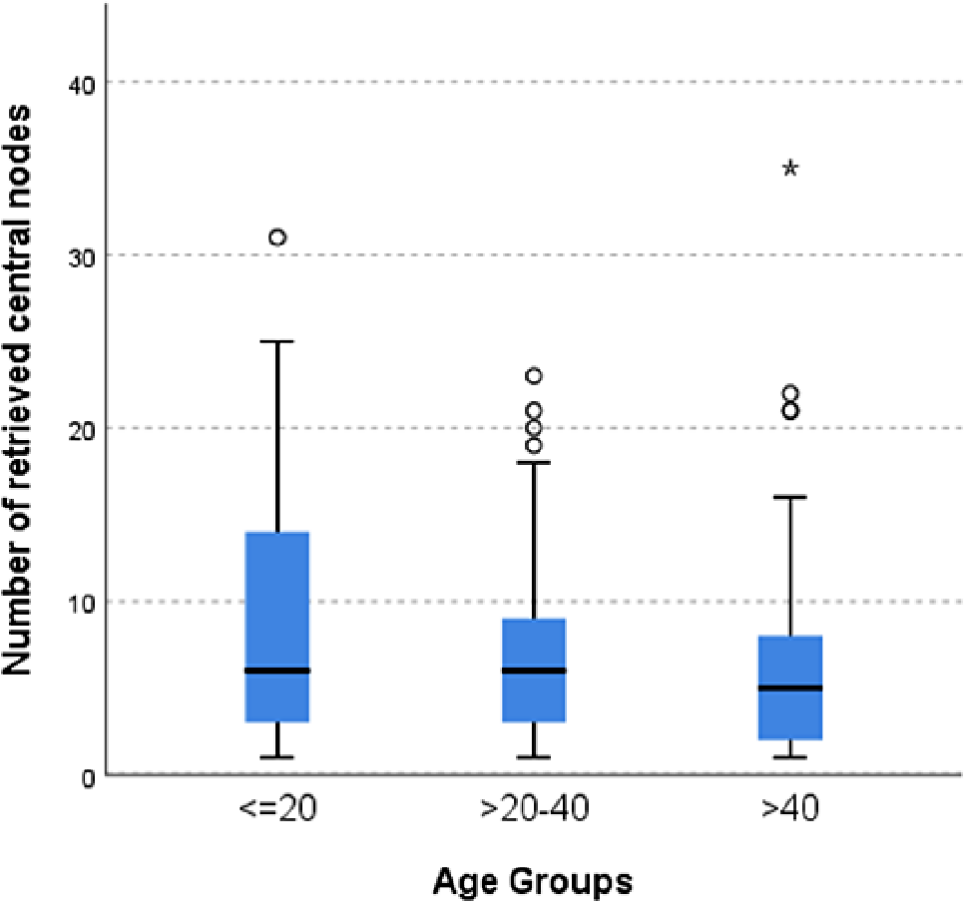
Box plot showing nodal harvest according to age groups

On the other hand, the lateral nodal harvest was significantly higher in the < 20 years group (*p* =.004) (Fig. 2) with no affection to the number of positive lateral nodes retrieved (*p* =.22).

**Fig. 3:**
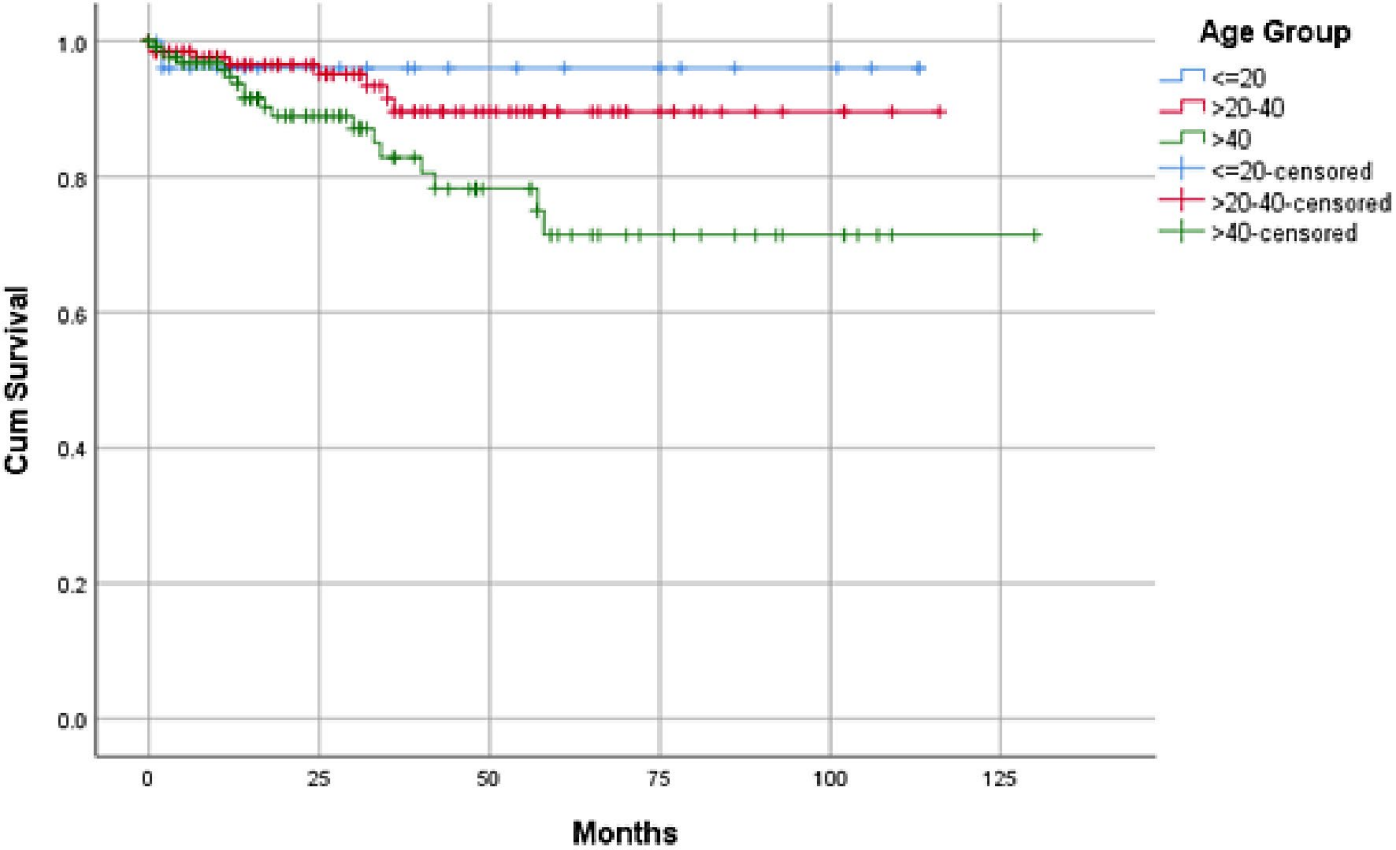
Kaplan-Meier curve showing disease-free survival according to age groups

The length of hospital stay was significantly longer in the < 20-year group (*p* =.013). Recurrence was very infrequent in group I (1/26 patients) (*p* =.026). In addition, the younger the age group the longer the disease-free survival (DFS) (estimated mean 108.6, 106.4, and 101.8, respectively) (*p* =.043) (Fig. 3).

## Discussion

PTC is the most diagnosed thyroid malignancy [11]. Fortunately, PTC is usually treatable and shows a good prognosis if diagnosed early although it is also accompanied by a high incidence of lymph node metastases [12]. Based on different studies, the 5 and 10-year survival rates of PTC Ethnic variation in thyroid cancer have received significant acknowledgment in the existing literature. Al-Ibraheem et al. stated that DTC had an excellent prognosis in Arab patients [13].

The optimal management for those patients is achieved by performing the most appropriate surgery at the time of diagnosis to offer the best prognosis and minimize the risk to those patients and the need for unnecessary secondary procedures [14]. Unfortunately, when the patients present without clinical cervical lymph node metastasis, both neck ultrasound and contrast-enhanced computed tomography have low sensitivity to detect CLN status [12].

Nowadays, most patients with a diagnosis of thyroid cancer are submitted to total thyroidectomy. A high proportion also receive CLND even in the absence of documented lymph node metastases [15]. In our study, all patients underwent total thyroidectomy and at least ipsilateral CLND. In addition, LLND was performed in 52.7% of patients with clinically and radiologically suspicious lateral LNs.

For patients with PTC, there is little direct evidence of clinical benefit in terms of lower recurrence or increased OS for thyroidectomy with prophylactic CLND compared to thyroidectomy alone [16, 17]. On the other hand, clear and consistent evidence demonstrates greater morbidity from neck lymph node dissection [18, 19]. Thus, surgeons should balance the oncologic benefit of lymph node dissection with the risk of surgical complications. In our study, the rate of postoperative hypocalcemia and RLN injury was in the lower range of the incidence found in the literature. The primary causes of postoperative hypoparathyroidism are direct injury, devascularization, or unintended excision of the parathyroid gland [20]. In a systematic review and meta-analysis of 115 studies evaluating the predictors of postoperative hypocalcemia, the median incidence of post-surgical hypocalcemia was 28% [21], compared to only 19% in our study.

The other main complication related to CLND is RLN injury. It is rare but potentially severe and life-threatening [22] In our study, RLN injury was observed in 9.2% of patients compared to 11.7% documented in a retrospective cohort study including 1547 patients [23].

Age is a major prognostic factor for the risk of LNM and recurrence in patients with PTC [24]. A previous meta-analysis demonstrated that age < 45 years with PTC may have an increased risk of LNM in clinical practice (pooled OR = 1.52). Even though age ≥ 45 years is usually associated with a poor prognosis and increased risk of recurrence, it was also reported that age < 45 years is a poor predictor of the prognosis of CLNM in PTC patients [12]. In this study, we found that the younger the age the higher the possibility of CLNM, however lateral LN status was similar in all age groups.

The results of our study show that younger patients with PTC are more likely than older patients to have CLNM. The risk of CLNM increased with decreasing age however the mean central node harvest was nearly equal in all groups. Younger age was not associated with a greater mean number of positive lymph nodes. Furthermore, patients aged < 20 years had nearly similar rates of lateral neck disease compared to other groups. Zhang et al. performed a single-institution retrospective study examining rates of lymph node metastasis in 1226 patients with papillary thyroid microcarcinoma. They compared patients aged < 39 years with those 40 to 59 years and ≥ 60 years. Like our study, they found that older patients were less likely to have lymph node metastases and high-volume lymph node metastases (> 5 positive nodes) [25].

Despite having a higher incidence of CLNM, DFS, and OS were longer and recurrence rates were lower in younger age groups regardless of the nodal status. In contrast, a retrospective study conducted on 81 patients younger than 17 years old with DTC showed favorable OS rates however patients who had initial lymph node metastases diagnosed before reaching puberty have a higher propensity for experiencing a greater number of disease events [26].

The main limitations of our study are that it is a retrospective study, a single-center experience in addition to the heterogenicity of the surgeon’s experience and heterogenicity of the surgical techniques for the patients ranging from ipsilateral CLND to bilateral CLND and bilateral LLND.

## Conclusion

CLND is associated with low morbidity, most commonly temporary hypocalcemia. About two-thirds of papillary cancers show positive central nodes. Radiology had a very low accuracy and sensitivity in detecting central node disease. Patients with spread to lateral node groups will almost always have a central nodal spread. Although patients aged below twenty years had a higher probability of harboring malignancy in central nodes and higher lateral node harvest on dissection, they do have a lower incidence of recurrence and longer DFS.

## Data Availability

All the clinical, radiological & pathological data used in this manuscript are available on the Mansoura University medical system (Ibn Sina Hospital management system). http://srv137.mans.edu.eg/mus/newSystem/.
